# High-tech, high-touch physical education: drone soccer as a sustainable sport activity for sport-marginalized students

**DOI:** 10.3389/fpsyg.2026.1787499

**Published:** 2026-04-09

**Authors:** Yongchul Kwon, Jinwoo Park, Gunsang Cho, Donghyun Kim, Minseo Kang, Dongsuk Yang

**Affiliations:** 1 Department of Physical Education, Pusan National University, Busan, Republic of Korea; 2 Department of LifeSports Guidance, Dongseo University, Busan, Republic of Korea

**Keywords:** drone soccer, high-tech, high-touch, inclusive participation, motivation, self-efficacy, sport-marginalized students, technology-integrated physical education

## Abstract

**Introduction:**

Digital technologies in school physical education (PE) may broaden participation and social interaction, particularly for students marginalized in traditional sport-based PE. However, qualitative evidence remains limited regarding how technology-based team sports such as drone soccer shape students' motivational and relational experiences and how teachers' pedagogical practices evolve during implementation. This study examined how a school-based drone soccer program shaped students' motivational and relational experiences in PE, with particular attention to sport-marginalized students. It also explored changes in teachers' pedagogical practices and beliefs.

**Methods:**

A qualitative multiple-case study design was employed to examine commonalities and contrasts across two school contexts. The drone soccer program was implemented as part of a district-led digital PE initiative in one girls' and one boys' middle school in Busan, South Korea. The program followed a standardized curriculum and was delivered over 6 weeks, with 1 weekly session consisting of two consecutive class periods (12 class periods total). Data were generated through semi-structured interviews with six purposefully selected students and three teachers, field observations, and document analysis, and were analyzed using inductive category analysis with cross-case comparison.

**Results:**

Across both schools, learning progressed from basic drone-control training to role-differentiated gameplay and preparation for an inter-school tournament, creating multiple participation pathways through differentiated roles. In the girls' school, initial anxiety and hesitant participation shifted toward supportive peer interaction and a student-led practice culture. In the boys' school, an initially competition-oriented climate evolved into more deliberate teamwork and strategy sharing. In both contexts, sport-marginalized students assumed core roles and reported increased engagement, enhanced perceived competence and self-efficacy, and stronger peer connections through sustained collaboration and shared goals. Teachers described an expanded instructional orientation toward technology-integrated and more inclusive PE, emphasizing role design, scaffolding, and team processes.

**Conclusions:**

Drone soccer appears to offer a feasible “high-tech, high-touch” team-based PE approach that may enhance motivational quality, belonging, and socially meaningful participation, particularly for sport-marginalized students, while supporting shifts toward more inclusive and technology-integrated pedagogical practices. Conceptually, the findings suggest that redesigning participation structures through role differentiation may broaden legitimate forms of contribution in school PE.

## 1 Introduction

Digital transformation is widely discussed as a structural shift that reshapes educational aims, content, and pedagogy, including the domains of sport and physical education (PE; Voogt and Roblin, 2012; Casey et al., 2017). Alongside calls for learners to develop integrated competencies such as digital literacy, problem-solving, collaboration, and creative thinking (OECD, 2018), PE has been encouraged to rethink how learning experiences are designed as digital technologies become part of everyday schooling (Jastrow et al., 2022; Tohănean et al., 2025). Importantly, the key issue is not technology adoption itself, but whether technology-integrated learning environments can improve the quality of participation and support students' motivational and relational experiences in PE.

This challenge is particularly salient for students who are sport-marginalized in conventional PE settings. In this study, we use the term sport-marginalized students as an analytic descriptor referring to students whose participation in conventional sport-based PE is persistently limited by low perceived competence, avoidance of performance-oriented activities, or social discomfort within ability-stratified classroom climates. We conceptualize marginalization not as an individual deficit but as a participation condition shaped by instructional structures and peer norms. This perspective also resonates with scholarship in inclusive physical education, where students with disabilities often encounter participation constraints shaped less by impairment itself and more by instructional structures, peer norms, and teachers' expectations. While sport-marginalization and disability are conceptually distinct, both highlight how participation inequities are socially and pedagogically constructed rather than inherently located within the individual.

Prior research suggests that perceived pressure regarding physical competence, low motivation, and socially patterned participation experiences such as gendered expectations can reduce willingness to engage in PE (Ennis, 2017; Flintoff and Scraton, 2001). In performance- and ability-oriented PE cultures, the social construction of “ability” and related hierarchies can widen participation disparities and reinforce exclusionary experiences for some students (Evans and Penney, 2008; Kirk, 2010). Recent scholarship on inclusion similarly indicates that participation inequities are often shaped by teachers' perceptions, classroom norms, and instructional structures rather than by individual deficits alone (Karamani et al., 2024; Ulset et al., 2025). From this perspective, broadening participation requires pedagogical designs that expand legitimate ways of contributing, strengthen perceived competence, and support peer connection and belonging within a supportive motivational climate (Ennis, 2017).

Technology-integrated physical activities have been discussed as one potential pathway for diversifying participation in PE (Jastrow et al., 2022; Tohănean et al., 2025). When digital tools are coupled with intentional pedagogy, students can participate through multiple modes that extend beyond physical performance, including manipulation, strategic thinking, coordination, and collaborative problem-solving (Mokmin and Rassy, 2024). This aligns with arguments that technology should operate as a mediator for interaction and relationship building, rather than as a replacement for learning (Godhe et al., 2019), and with evidence that learning designs supporting social and teaching presence can enhance peer interaction and learning quality (Martin et al., 2022). In PE contexts, cooperative learning research also provides a useful lens, as structured interdependence and role clarity can promote meaningful participation and social learning for diverse students (Bores-García et al., 2021; Casey and Goodyear, 2015), while studies on digital technologies within cooperative learning highlight both barriers and enabling conditions for sustained classroom use (Bodsworth and Goodyear, 2017; Luptáková and Antala, 2017).

Within this broader landscape, drone-based sport offers a distinctive case of digitally mediated team activity. Competitive drone racing emphasizes visual processing, control precision, spatial awareness, and context-sensitive decision making, rather than high levels of strength or endurance (Pfeiffer and Scaramuzza, 2021). Drone soccer, in particular, is organized around differentiated team roles operating drones in a bounded space and requires tactical communication, coordination, and shared strategy. Its educational potential has been discussed in relation to scaffolded skill development and systematic role differentiation (Noor and Faziehan, 2025; Techanamurthy et al., 2024), and the program examined in this study was aligned with formal rules and competition formats developed by the Federation of International Dronesoccer Association (FIDA) (2025). These features suggest that drone soccer may create alternative participation pathways for students who experience pressure or disengagement in conventional sport formats by enabling multiple entry points to competence and contribution.

The drone soccer program analyzed in this study was implemented as part of a district-led initiative that provided equipment, specialist support, a standardized curriculum, and organized competitive opportunities. Such implementation supports matter because teachers' pedagogical beliefs and perceived competence in technology integration shape what is enacted in practice (Ertmer and Ottenbreit-Leftwich, 2010; Saiz-González et al., 2025), and professional learning related to digital pedagogy can influence teacher self-efficacy and wellbeing (Shi et al., 2025). In inclusive PE, teachers' confidence and efficacy are also closely linked to how participation opportunities are designed and sustained for diverse learners (Antala et al., 2022). Despite growing research on digital technologies in PE (Jastrow et al., 2022; Tohănean et al., 2025) and inclusion-oriented practice (Karamani et al., 2024; Ulset et al., 2025), relatively little is known about how policy-supported digital sport programs are enacted across contrasting school contexts and gender compositions. Few studies have examined how such initiatives reorganize participation structures in ways that address motivational and relational inequities within everyday school PE. It is also unclear how such enactments shape students' motivational, relational, and socio-emotional experiences, particularly for sport-marginalized students. Understanding how these initiatives operate in practice is essential not only for evaluating short-term engagement but also for assessing their potential sustainability within school systems.

Accordingly, this qualitative multiple-case study investigates how a district-led drone soccer program was designed and implemented in one girls' and one boys' middle school in Korea. It also examines how participation in drone-based PE shaped students' motivational and socio-emotional experiences, with particular attention to sport-marginalized students, and how participating teachers' instructional practices and pedagogical beliefs evolved during implementation. By foregrounding motivational and relational processes such as engagement, perceived competence, self-efficacy, and peer connection (Ennis, 2017; Bores-García et al., 2021; Antala et al., 2022), the study aims to extend understanding of how digitally mediated, team-based PE activities can broaden meaningful participation and inform the design of inclusive technology-integrated PE in school systems.

## 2 Research design and methods

### 2.1 Research design

This study adopted a qualitative multiple-case study design to examine how a district-led drone soccer program was enacted in school physical education (PE) settings and how students' participation and socio-emotional learning experiences were constructed through implementation. We conducted a comparative analysis of two contrasting school contexts, one girls' middle school and one boys' middle school, to explore how the same program was taken up in practice and how contextual conditions shaped participation structures, interactional patterns, and learning cultures.

The focal program was delivered as part of a district initiative (“Digital-Based Physical Education Promotion Project”) led by the Busan Bukbu Office of Education under the Busan Metropolitan City Office of Education. The program followed an identical, standardized curriculum delivered once a week for two consecutive class periods over 6 weeks (six weekly sessions; 12 class periods in total). Specialist instructors dispatched by the education office facilitated the weekly sessions, while school PE teachers supported implementation by recruiting students, supervising safety, and assisting with operational arrangements. After completion of the sessions, students from both schools participated in an interschool drone soccer tournament organized by the education office, providing a shared culminating event that further illuminated participation dynamics and team-based learning processes.

The two cases were purposefully selected because they shared an identical policy-supported program structure while differing in school culture and student composition (girls' school vs. boys' school). This design enabled an in-depth and contextualized understanding of how a technology-mediated, team-based activity can shape students' engagement and peer interaction, and how implementation features and local school norms may contribute to similarities and differences in participation experiences across settings.

### 2.2 Participants

The district-level drone soccer program was implemented in eight middle schools; however, this study focused on two case schools. A total of 10 students participated in the drone soccer program at these two schools, with five students from each school (School A, *n* = 5; School B, *n* = 5). Students were recruited for the program through voluntary sign-up at each school, with PE teachers informing students about the opportunity and encouraging participation among those who had shown recurrent passive participation or low perceived confidence in regular PE classes. Six students consented to participate in individual interviews, and we used purposive sampling to recruit these six student interviewees (three girls and three boys) and three teacher interviewees for in-depth qualitative interviews.

We used purposive sampling to ensure representation across both schools and genders, prioritizing students whom the PE teachers had identified as showing recurrent avoidance or passive participation and/or low perceived competence in regular PE. Identification relied primarily on sustained observations by the PE teachers in regular PE contexts and was corroborated by early-session participation patterns in the drone soccer program. All students completed the 6-week program, and no participants were excluded due to withdrawal. The term “sport-marginalized” was used as an analytic descriptor rather than a diagnostic label.

All interviewed students were in Grades 8–9 (approximately 14–15 years old). Students from School A reported no prior experience with drone operation, whereas some students from School B had previous exposure to drone-related or coding activities. Two PE teachers from the focal schools were directly involved in program implementation and student supervision. To capture complementary perspectives on implementation conditions and transferability across schools, we also interviewed one teacher from another middle school that had participated in the same district initiative. Although this teacher did not work at either focal school, they provided practice-based insights into program delivery and practical considerations for adoption in other school settings. To protect participants' confidentiality while distinguishing between speakers, interview participants are referred to using pseudonymous codes (T1–T3 for teachers and S1–S6 for students).

### 2.3 Data collection

Data were collected over approximately 2 months, spanning the full 6-week implementation of the drone soccer program and the subsequent interschool tournament organized by the district education office. A qualitative, multi-source data collection strategy was used, drawing on field observations, document analysis, and semi-structured interviews.

Documentary data included the program implementation plan and teaching and learning guides provided by the education office, lesson logs and student activity records produced by teachers at each school, and the tournament participation plan and post-event report. These materials were used to examine program planning and enactment processes and to identify formally documented patterns of student participation and instructional activity.

Semi-structured interviews were conducted with six purposively sampled students and three teachers. Student interviews were conducted individually and focused on experiences prior to and during participation, reasons for engagement, and perceived changes in confidence, learning-related understanding, and peer interaction. Teacher interviews explored instructional planning and in-class decision making, implementation challenges, and teachers' observations of changes in students' engagement, participation patterns, and interpersonal dynamics. To triangulate perspectives on implementation conditions and transferability across schools, an additional interview was conducted with a teacher from another school that had participated in the same district initiative. All interviews lasted approximately 30–60 min, were audio-recorded with consent, and were transcribed verbatim.

Observational data were generated through participant observation of lessons, practice sessions, and tournament scenes. Across sessions, the researcher systematically recorded students' participation dispositions, interactional patterns, collaborative behaviors, teacher–student interactions, and the overall instructional climate. Field notes provided detailed descriptions of salient episodes during lessons, practice, and tournament participation, including student utterances, non-verbal behaviors, within-team dynamics, and moments of challenge or change.

### 2.4 Data analyses

Data analysis was guided by inductive category analysis and proceeded iteratively alongside data familiarization. We first read interview transcripts, observation records (lessons and practice sessions), and documentary materials multiple times to identify meaning units relevant to the research questions. Meaning units were then condensed and grouped into subcategories by clustering statements and episodes that shared similar content and contextual features. Through a stepwise analytic process, we compared and refined subcategories, examined relationships among them, and integrated them into higher-order categories. Coding and data management were conducted in Microsoft Excel to organize excerpts, track category development, and facilitate constant comparison across sources.

A cross-case comparison was conducted between the girls' and boys' school datasets to identify shared implementation processes and context-specific variations in participation structures, interactional patterns, and learning cultures across the program and tournament preparation. Particular attention was given to how digitally mediated, team-based activities shaped the motivational and socio-emotional experiences of students who were less engaged or typically underserved in conventional PE contexts. To address this aim, we repeatedly compared meaning units drawn from student and teacher accounts and observation records, focusing on shifts in engagement and perceived competence, changes in peer interaction and collaboration, and how role differentiation and team processes supported participation.

To enhance trustworthiness, we used triangulation across data sources (documents, interviews, and observation notes), conducted member checking of key interpretations and category labels, and engaged in peer debriefing with a colleague researcher. Throughout the analytic process, the researcher maintained a research journal and reflexive memos to document analytic decisions and to examine how prior assumptions might shape interpretation, thereby supporting transparency and analytic coherence.

Ultimately, the codes were clustered into subthemes (Table 1) and integrated into two overarching themes: “Implementation Process of the Drone Soccer Program” and “Perceived Outcomes of the Drone Soccer Program.”

**Table 1:** Coding framework for the drone soccer program.

Codes	Subthemes	Themes
Lessons integrating conceptual understanding and hands-on practice Progressive scaffolding of task difficulty across sessions Co-teaching and coordination between district instructors and school PE teachers	Common instructional trajectory	Implementation process of the drone soccer program (RQ1)
Girls' school: shifting from anxiety to confidence Girls' school: formation of a practice/play culture through continuous training Boys' school: competitive participation climate Boys' school: recognizing the need for cooperation through tournament preparation Across schools: sport-marginalized students participating consistently across sessions	Differences in participation patterns by school	
Voluntary extra practice for tournament preparation Increased engagement through extra practice Continuity of participation from regular classes to tournaments and then to clubs	Expansion of learning through tournament participation	
Previously passive students becoming actively engaged in drone sessions Development of piloting skills and self-efficacy through repeated practice Enhanced self-directedness through autonomous practice	Learners' internal changes and active participation	Perceived Outcomes of the drone soccer program (RQ2)
Increased real-time communication during matches (directions, cheering, feedback) Interdependence and complementarity supported by role differentiation Sense of belonging as “a real team” shaped through tournament experiences	Strengthening of cooperation and communication	
Reframing PE as learning involving strategy, technology, and collaboration Teachers' efforts to adapt, reorganize, or extend technology-integrated PE activities Recognition of the need for ongoing support (expertise, equipment, staffing)	Reconfiguration and expansion of technology-based PE practice	

As shown in Table 1, initial codes were progressively refined and integrated into subthemes and overarching themes through iterative comparison. For example, statements indicating that students “initially felt anxious but gradually gained confidence” were coded as “girls' school: shifting from anxiety to confidence” and later grouped under “Differences in participation patterns by school.” Deviant cases, including students who initially struggled with technical control demands, were also examined during analysis.

### 2.5 Researcher positionality and reflexivity

The researcher served as a supervising PE teacher at one of the participating schools (School A) and was directly involved in program implementation, student observation, and data collection. Thus, the researcher occupied a dual role as practitioner and participant observer. Although this position enabled close, context-sensitive understanding of the implementation process, it also raised potential concerns regarding power asymmetry and interpretive bias.

To minimize potential power imbalances, the researcher clarified to students that he was not their classroom instructor and did not evaluate their grades. Students were explicitly informed that their interview responses would not affect grades or teacher evaluations. Open-ended questions were used to encourage candid reflection. Although the use of an external interviewer was considered, the researcher conducted the interviews due to contextual familiarity, while reflexive memoing was employed to bracket prior assumptions. Critical or negative perspectives (e.g., initial frustration with technical control demands) were retained in the analysis to avoid overly positive interpretations.

Reflexive practices were integrated throughout the study. The researcher maintained a research journal and reflexive memos to document analytic decisions and potential assumptions during data collection and analysis. Interpretations were triangulated across interviews, observations, and documentary records, and were cross-checked with a teacher from another participating school. In addition, peer debriefing with a colleague researcher was conducted to enhance analytic transparency and consistency.

### 2.6 Ethical considerations

All procedures were conducted in accordance with established ethical guidelines for research involving human participants. Prior to data collection, participants were informed of the study purpose and procedures, the voluntary nature of participation, confidentiality protections, and their right to withdraw at any time without penalty. Written informed consent was obtained from all adult participants. Because student participants were minors, written consent was obtained from legal guardians and students provided assent prior to participation. Participants were also informed that participation or non-participation would not affect grades, evaluations, or access to school activities.

Interviews were audio-recorded with permission and transcribed verbatim. All materials were de-identified during transcription by removing personal identifiers and assigning pseudonymous codes (e.g., T1–T3, S1–S6). Data were used solely for research purposes, stored securely, and accessed only by the research team. These safeguards were implemented to minimize potential risks and to protect participants' rights throughout the research process.

## 3 Results

### 3.1 Implementation process of the drone soccer program

This section describes how the district-led drone soccer program was implemented across the two participating schools. Drawing on interviews, observations, and program documents, we first outline the shared sequence of instructional activities and operational arrangements and then highlight school-specific differences in participation patterns and learning cultures. We also describe how tournament participation extended learning beyond the formal sessions and shaped subsequent engagement.

#### 3.1.1 Shared instructional sequence and operational arrangements

The program was delivered as part of the “Digital-Based Physical Education Promotion Project” administered by the district education office and was implemented in eight middle schools, including the two schools examined in this study (one girls' middle school and one boys' middle school). Each school recruited five student participants. A specialist drone instructor and an assistant instructor collaborated with school teachers to deliver the after-school sessions. The program consisted of six weekly sessions, each scheduled as two consecutive class periods (approximately 2 h), for a total of 12 class periods.

Session 1 (Periods 1–2) focused on orientation, foundational concepts of drone soccer, and basic control skills. Students practiced core flight procedures using Attitude mode (ATTI), including takeoff and landing, forward/backward/left/right movement, and return flight. Using an entry-level drone and a simple obstacle course, they developed basic operational skills such as maintaining safe distance, regulating speed, and stabilizing the drone's posture.

Session 2 (Periods 3–4) introduced Stabilize mode with an auto-leveling function to support control accuracy. Through tasks such as angle correction, maintaining hover, and passing through obstacles, students practiced fine adjustments and spatial awareness.

From session 3 (Periods 5–6), instruction shifted from control-focused practice to game-based preparation. Students learned to operate drone soccer–specific equipment and manage the drone system (e.g., propeller inspection, battery management, and emergency stop procedures). Practice took place on a court configured to approximate competition conditions to support game readiness.

Session 4 (Periods 7–8) differentiated team roles (pilot, assistant, observer). Teams participated in short scrimmages guided by predefined performance criteria, and instruction addressed tactical decision making (e.g., maintaining a defensive line, securing offensive routes, and judging flight paths under dynamic conditions).

Session 5 (Periods 9–10) involved a joint session between the two schools. Using the same drones and equipment, participants conducted simulated matches under official youth drone soccer rules. Through practice with students from another school, they tested performance under conditions similar to real match situations.

Session 6 (Periods 11–12) emphasized competition-oriented rehearsal. Students were assigned offensive and defensive roles and participated in 1:1 and 2:2 scrimmages to approximate tournament conditions. They applied spatial awareness and tactical decision making while strengthening within-team communication. The latter part of the session was used to review and adjust tactics and role performance based on gameplay experiences.

Across sessions, a court was installed at the center of the indoor gymnasium, and flight and waiting routes were separated to ensure safe movement. Safety procedures were reinforced in every session, including establishing no-fly zones, maintaining spectator safety distances, and repeatedly demonstrating emergency stop procedures. At the end of each session, teams engaged in structured reflection, sharing operational difficulties, gaps in strategy execution, and collaboration approaches. Some teams reviewed recorded videos to check timing and defensive positioning and incorporated these insights into subsequent sessions.

**Figure 1:**
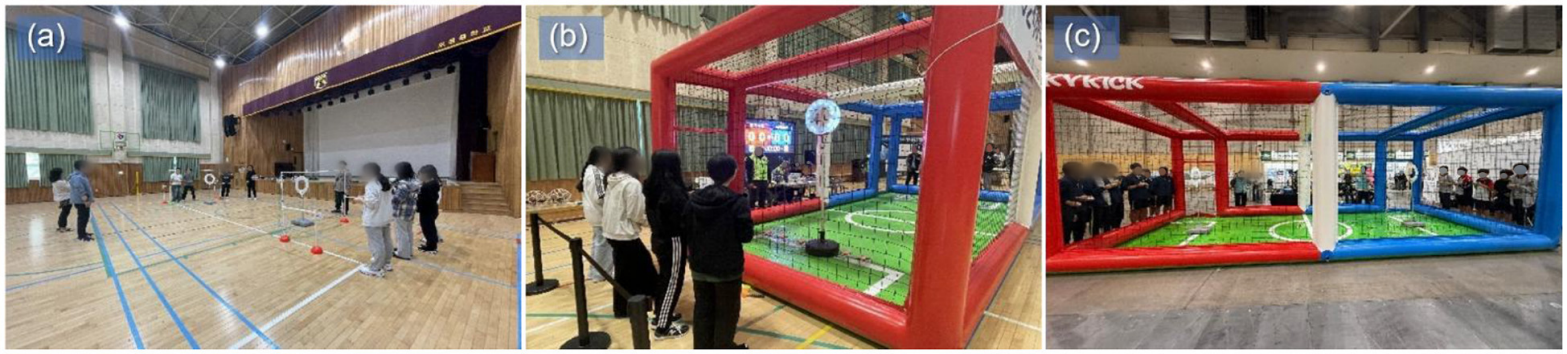
Scenes from the joint practice session and the drone soccer tournament involving School A and School B. **(a)** Joint practice session involving both schools; **(b)** School A during the tournament; **(c)** School B during the tournament.

After the formal program ended, both schools continued voluntary practice, and participating students competed in a drone soccer tournament hosted by the district education office. This provided opportunities for the skills and strategies developed during the sessions to be enacted in an external competitive context. Figure 1 presents scenes from the formal program and the drone soccer tournament.

#### 3.1.2 Differences in participation patterns by school

Although both schools implemented the same drone soccer program, students' participation patterns differed across settings. School A (girls' middle school) included students with little prior experience operating drones. In early sessions, several students adopted a reserved stance, often describing difficulty with control and hesitation about active involvement. Across repeated practice, however, students described growing confidence and increasingly coordinated, team-based play. Drone activities also extended into voluntary practice beyond formal sessions (e.g., during breaks, lunchtime, and after school), and an informal peer culture formed around continued practice. As the program progressed, students became more actively involved in role allocation, peer feedback, defensive coordination, and strategy planning.

“At first, they lacked confidence because they thought they would not do well, but an atmosphere developed where they felt it was okay to work together with friends even if their skills were not strong. As their interest in drone soccer grew, a student-led practice culture emerged where they practiced drones during breaks, lunchtime, and after school.” (T2)

“At first, the drone was so difficult that I just watched from the back. But my team said, ‘Let's plan the strategy together instead of you piloting,' and that is when the class became fun.” (S1)

In contrast, School B (boys' middle school) included several students with prior exposure to drones or related coding activities. From the outset, participation was characterized by relatively high technical proficiency and confidence and a more competitive climate, with individual performance (speed and precision) frequently emphasized during scrimmages. Over time, particularly during tournament preparation and practice matches against other schools, students increasingly recognized the practical necessity of teamwork. Team-based practice and strategy sharing became more active as students worked to improve collective performance.

“Many students could handle drones skillfully from the beginning. At first, competition was strong and there was a lot of individual play, but as they prepared for the tournament, they realized on their own that cooperation mattered.” (T2)

“Each of us thought we were good, but we kept losing, so we realized you cannot do it alone.” (S4)

These observations suggest that even with an identical curriculum, program enactment and participation patterns can vary depending on students' prior experiences and group composition. Participation cultures that formed organically within each school and the ways students interacted within teams continued to shape subsequent implementation and engagement patterns. Notably, in both schools, some students who were typically passive or had low participation in regular PE consistently engaged in drone soccer across all sessions and assumed central roles such as pilot or strategy lead. Overall, these patterns appeared to be associated with prior experience and peer culture rather than gender alone, indicating that the same technology-based instruction may be taken up in different ways depending on school context.

#### 3.1.3 Tournament participation and the expansion of learning

Even after the formal program ended, students at both schools continued voluntary practice on their campuses, using breaks and after-school time to rehearse drone control and gameplay. Both schools then participated in a drone soccer tournament hosted by the district education office.

The tournament was an official event for local middle schools and was held as a large-scale indoor competition centered on a gymnasium court. It included match announcing and event staging using lighting and sound equipment. As students attempted to apply what they had practiced in class against unfamiliar schools, they described heightened immersion and tension compared with routine school-based practice.

School A (girls' middle school) entered as the only all-girls team. Although their technical proficiency was described as relatively lower, they maintained consistent play across matches by relying on teamwork and strategic communication. Through a stable, defense-oriented approach, they placed third.

“We lost some matches, but I was proud that we made it through to the end as a team and won third place. I thought we would automatically lose because they were boys, but I did not feel any gender difference at all. When we beat a boys' team, I was really excited.” (S1)

During tournament preparation and participation, students encouraged one another and co-developed strategies, and teachers described a more positive team climate. Compared with practice sessions, students described greater confidence and a sense of belonging in a more authentic competitive setting. After the tournament, students organized a club and continued practicing drones during breaks and after school.

School B (boys' middle school) advanced to the top tier from the preliminary rounds, drawing on strong technical skills and effective tactical execution. During matches, individual techniques and within-team coordination were described as relatively stable. After the tournament, some students continued drone soccer as a self-directed club activity and maintained practice outside regular class time. Notably, tournament preparation and participation were accompanied by a shift from an initially competitive, individual-centered stance toward clearer role differentiation and more cooperative play. Students also described more proactive preparation for unexpected situations, including those related to equipment malfunction.

“The tournament became an opportunity for students to apply what they had learned in real situations and to confirm the importance of cooperation.” (T2)

“I thought our skill level was much better, but once we lost because the drone suddenly crashed. After that, we prepared for situations like that at any time, and when something similar happened again, we were able to handle it wisely.” (S6)

Overall, students applied skills and strategies developed during the sessions in match settings and continued self-directed practice and team-based activities afterward. Tournament participation served as a context that extended school-based training into an external arena of practice. In both schools, students expressed disappointment that the program had ended and formed student-led drone soccer clubs to sustain engagement beyond regular class time, suggesting that drone-based activities can be voluntarily maintained and expanded outside formal instructional settings.

### 3.2 Perceived psychological, social, and pedagogical outcomes

This section reports students' and teachers' accounts of outcomes associated with participation in the drone soccer program. Across both schools, students described greater confidence and sustained engagement, along with strengthened teamwork and peer interaction. Teachers described technology-integrated PE as providing additional pathways for participation and as prompting reflection on conventional performance-centered structures in PE, particularly for students who were typically less engaged in regular PE.

#### 3.2.1 Increased confidence and sustained engagement

The drone soccer program provided opportunities for participation for students who reported low confidence in conventional PE or tended to avoid engagement. In particular, students who described anxiety about athletic ability or initially displayed reserved participation reported growing self-efficacy through repeated practice and role differentiation within their teams. This pattern was described most prominently among students in the girls' middle school.

By piloting drones and taking responsibility during gameplay, students described deep engagement, a sense of accomplishment, and increasing confidence. Some students noted that sustained concentration and repeated practice shifted participation from “completing a task” to “an activity they wanted to do.” As students came to believe that they could successfully operate the drone, they described this belief as developing into greater self-efficacy. Notably, some students who had previously participated minimally in regular PE attended every session without being late, suggesting increased willingness to participate and sustained engagement.

“I have a personality that is not very outgoing, so I participated passively in PE. But with drones, I thought, ‘I want to try it at least once.' After practicing a few times, I felt that I could do it, too.” (S2)

“At first, I worried that drone control was too difficult and that I might not be able to do it well, but as I kept practicing, I gained confidence that even if I made mistakes, I could just try again.” (S3)

Students also described that, even with limited technical skill, they could contribute meaningfully as core team members, and match experiences against unfamiliar opponents were accompanied by further increases in confidence. Teachers similarly reported noticeable shifts in students' attitudes toward lessons, including increased initiative and more active participation.

“When I saw a student who struggled with physical activity come out first to prepare the drone for class, I could tell their desire to try it themselves had grown a lot.” (T3)

“A student who always said, ‘I can't do it' whenever PE started showed enough confidence to explain drone control methods to friends.” (T1)

Participation also extended beyond formal sessions into voluntary practice. As students encountered mistakes and failures, they described persisting through trial and error and practicing independently to improve technical skills, which was associated with a more self-directed learning stance. Voluntary practice during non-class time (e.g., upon arrival at school, during lunch, and after school) became part of some students' routines, and several students sustained their engagement by forming a drone club after the program ended.

For some students, drone operation also functioned as a channel for self-expression and a pathway to rebuilding confidence. Competencies that were less visible in conventional physical activities (e.g., control precision, situational judgment, and strategic contribution) became salient through the technological medium of drones. Taking responsibility for team strategy or guiding gameplay supported more positive self-perceptions within the activity.

In sum, students' accounts indicated a range of individual-level experiences associated with participation, including sustained engagement, immersion, increased self-efficacy, opportunities for self-expression, and strengthened self-directed practice. Over time, students described reduced hesitation about participation and greater willingness to take responsibility within team roles.

#### 3.2.2 Strengthened collaboration and communication

Because the drone soccer program was inherently team-based, students accumulated experiences throughout the lessons involving strategy development, role allocation, collaborative decision making, and feedback exchange. Across accounts and observations, these processes were associated with peer relationship building and interaction patterns that differed from those observed early in the program.

In the initial sessions, role performance within teams tended to be somewhat formal or procedural. Over time, however, students described more spontaneous communication and coordination in response to changing game situations. By observing one another's movements and exchanging situation-specific directions and encouragement, students increasingly framed their roles as integral to the team's overall strategy.

“Even if one person is good, it does not matter. We have to move together to score.” (S1)

“At first, we were quiet, but during matches we talked and cheered so much that our throats hurt.” (S2)

These collaborative interactions were described not only as ways to improve match performance but also as opportunities to support one another while pursuing shared goals. Mistakes and misjudgments during gameplay often led to moments requiring negotiation and adjustment, and over time, team communication was described as becoming more stable and grounded in trust.

Students also described that drone soccer emphasized skill acquisition and strategic thinking and, in doing so, reduced the salience of differences related to physical strength. For example, students noted that “It felt like skill matters more than gender or strength. Anyone can do it,” and that “Even if your technical skill is not strong, the team can win if roles are divided well. Sometimes helping is more important.” These remarks suggest that students increasingly recognized how different abilities could be combined and valued within a team.

As the program progressed, students described a stronger sense of mutual trust and responsibility through role switching, sharing tactics, and post-game feedback discussions, contributing to a more community-oriented team culture. Some students sustained engagement after the program by forming clubs and continuing team-based practice during lunch and after school. Following the tournament, students expressed a desire to continue the activity (e.g., “I hope we have drone classes next semester, too,” and “Preparing for the tournament made us feel like a real team. I want to keep doing it together.”).

In sum, students' accounts indicated that the drone soccer program supported collaborative engagement in shared tasks and was accompanied by relational processes such as communication, role-based responsibility, and peer support. Beyond technical learning, the activity functioned as a context in which peer relationships were negotiated and sustained over time.

#### 3.2.3 Shifts in teachers' perspectives and the extension of practice

Drone soccer lessons were described by physical education teachers as opening new possibilities for technology-integrated instruction. At the outset, some teachers expressed skepticism due to the complexity of operating equipment, concerns about safety incidents, and unfamiliarity with technical control. As the program progressed, teachers described students' immersion and active participation as influencing how they viewed the instructional value of drone soccer.

Teachers reported that students who had been less engaged in conventional PE participated more actively when drones served as the central medium of instruction. They described technology as functioning not merely as a supplementary tool but as a mediator through which participation could be deliberately structured and engagement supported. Because lessons emphasized strategic thinking, operational skill, and collaboration alongside physical activity, teachers also noted that students with diverse backgrounds appeared to participate in and approach PE differently than in typical sport-centered formats.

Teachers further described that drone soccer highlighted the educational value of exploring and applying sport strategies and technical skills, rather than limiting PE to “moving the body.” In this sense, drone soccer was perceived as expanding PE toward learning experiences that integrate technological, cognitive, and social dimensions.

“It feels like drones expanded the boundaries of physical education. Even students who find physical activity difficult can experience ‘I can do this,' and because students are immersed on their own, it actually became easier to manage the class.” (T2)

“I started to think that PE is not only about physical activity, but also about understanding and applying strategy and skill.” (T3)

Building on these experiences, some teachers reported intentions to continue related practices after the program ended by launching school-based drone clubs or planning interdisciplinary PE lessons using drones. Teachers described increased interest in project-based lessons and student-led activities, and some noted that discussions emerged about linking technology, PE, and career exploration.

“This program made me realize that inclusive lessons using technology are possible in PE, too. I think I found some clues for supporting students who are reluctant to participate in physical activity.” (T1)

“Students asked to do it again next year, so we decided to recruit additional students who want to participate. I felt it should not be a one-time experience, but something we continue.” (T2)

Overall, teachers' accounts suggested that drone soccer extended beyond a short-term experience and was accompanied by changes in how teachers framed PE and participation. These accounts also indicated tangible follow-up practices, such as after-school clubs and interdisciplinary instruction, through which schools sought to sustain students' engagement beyond the formal program.

## 4 Discussion

### 4.1 Technology-integrated physical education and the inclusive participation of sport-marginalized students

The findings suggest that drone soccer can support more inclusive participation in physical education (PE) by offering alternative participation pathways for students who feel underserved in conventional PE formats. In performance- and ability-oriented PE cultures, participation can be shaped by hierarchies of “ability,” which may marginalize students who experience pressure about physical competence or report low willingness to engage (Ennis, 2017; Evans and Penney, 2008; Kirk, 2010). Recent scholarship also emphasizes that participation inequities are often co-produced through classroom norms and instructional structures rather than individual deficits alone (Karamani et al., 2024; Ulset et al., 2025). In this study, the girls' middle school case shows how students with no prior drone experience moved from initial hesitation to greater confidence through repeated practice and team-based gameplay, indicating that technology-mediated PE may broaden what counts as meaningful participation beyond physical performance.

Although drone soccer does not involve sustained moderate-to-vigorous activity comparable to endurance sports, it engages important dimensions of physical competence. Broader definitions of physical education include motor coordination, spatial awareness, and perceptual-motor control beyond energy expenditure alone. Drone piloting requires fine motor adjustments, visuomotor coordination, and real-time spatial judgment, suggesting a reconfiguration rather than a reduction of physical engagement.

This interpretation aligns with research arguing that the central issue is not technology adoption itself but whether technology-integrated learning environments reshape participation conditions and learning experiences in PE (Casey et al., 2017; Jastrow et al., 2022; Tohănean et al., 2025). While Noor and Faziehan (Noor and Faziehan, 2025) discussed drone soccer primarily in a STEM context, they similarly noted its potential to integrate technical skill development, role differentiation, and collaborative learning. Extending this line of work into PE, the present study provides qualitative evidence that a systematically designed digital sport format may reduce participation barriers when implemented as an inclusive lesson design attentive to learners' characteristics and participation conditions (Karamani et al., 2024; Ulset et al., 2025; Mokmin and Rassy, 2024; Baena-Morales et al., 2021). Importantly, the inclusive potential observed here appears to stem less from novelty and more from how the activity reorganized participation into multiple entry points (e.g., piloting, strategizing, observing, coordinating) through which students could experience competence and contribution within the team.

These participation shifts can be interpreted through Self-Determination Theory (SDT), particularly in terms of perceived competence and relatedness (Deci and Ryan, 2000; Ryan and Deci, 2017). Students' growing confidence and sustained engagement suggest that role differentiation supported competence experiences while team-based interaction fostered relational connection. Moreover, the movement from individual competition toward coordinated teamwork aligns with Achievement Goal Theory (AGT; Nicholls, 1989; Ames, 1992), indicating a shift toward more mastery-focused participation. From this perspective, drone soccer may recalibrate the motivational climate of PE in ways that support more inclusive engagement.

Interview accounts illustrate how participation criteria shifted in practice. For instance, a student in the girls' middle school noted, “At first, I only watched from the back, but the class became fun when I took on a strategy role,” suggesting that engagement was not contingent on athletic performance alone but could be supported through role-based contribution to team coordination and decision making. This pattern resonates with cooperative learning perspectives in PE, in which structured interdependence and role clarity can expand legitimate ways of contributing and support meaningful participation (Casey and Goodyear, 2015; Baena-Morales et al., 2021). In the present study, role differentiation appeared to operate as a practical mechanism through which students who were initially hesitant could participate meaningfully, build confidence through repeated involvement, and remain engaged as team demands became clearer.

The findings suggest that drone soccer may create contexts in which gendered expectations are less pronounced, as operational skill, strategy, and communication are emphasized over strength-based performance. Gendered participation experiences have long been shown to influence engagement in PE (Ennis, 2017; Flintoff and Scraton, 2001). In this study, a student from the all-girls team reported not strongly perceiving gender differences when competing against boys, suggesting that technology-mediated team activities may foreground skill and coordination in ways that support alternative participation pathways. However, given the limited scope of the sample, this observation should be interpreted cautiously and explored further in future research (Karamani et al., 2024; Ulset et al., 2025).

Taken together, drone soccer was described as a pedagogical mediator through which PE participation could be organized around multiple forms of competence and contribution, potentially supporting more inclusive motivational and relational experiences for sport-marginalized students (Casey et al., 2017; Jastrow et al., 2022; Tohănean et al., 2025). From a broader equity-oriented perspective, such participation pathways are consistent with practice-based approaches that connect PE to inclusive aims in education and wellbeing (Ulset et al., 2025; Baena-Morales et al., 2021; UNESCO, 2015).

These findings may also offer conceptual insight for inclusive PE contexts involving students with disabilities. Prior research indicates that participation inequities in inclusive settings are often shaped by instructional design, peer norms, and teachers' expectations rather than impairment alone. In this regard, the role-differentiated and digitally mediated structure of drone soccer illustrates how participation criteria can be reorganized to accommodate diverse competence profiles. Although the present study did not specifically examine students with disabilities, the reconfiguration of participation into multiple entry points may inform inclusive pedagogical approaches that seek to expand legitimate forms of contribution within mixed-ability classrooms.

### 4.2 Reconfiguring high-tech, high-touch collaboration and relational experiences through drone soccer

The findings suggest that the drone soccer program was associated with shifts in students' collaboration and communication, supporting learning experiences that emphasized relational processes. Within the team-based game structure, students repeatedly engaged in strategy development, role allocation, and real-time feedback, which fostered interdependence and shared responsibility. In this sense, the program functioned as a context for social learning that extended beyond technical skill acquisition. Across sessions, students' spontaneous exchanges of directions, adjustments following mistakes, and peer encouragement during gameplay indicate that interaction increasingly centered on team-based coordination rather than individual performance.

This interpretation aligns with evidence that cooperative learning can support relationship building and social learning in physical education (Bores-García et al., 2021; Casey and Goodyear, 2015). Research further suggests that when cooperative learning is combined with digital tools, peer interaction and joint task engagement can be strengthened, although sustained use depends on classroom conditions and pedagogical design (Bodsworth and Goodyear, 2017; Luptáková and Antala, 2017). In the present study, students' statements such as “It does not matter even if one person is good” and “We have to move together” suggest that they increasingly framed PE as a collaborative space oriented toward shared goals rather than as a stage for individual performance. This implies that role differentiation and collective aims can shift participation criteria from valuing “individual skill” to valuing “team contribution.”

A particularly notable pattern was that, as the technology-mediated activity was coupled with role-based instructional design, an initially individual-skill-centered and competition-driven participation structure (especially evident early in the boys' school case) appeared to be recalibrated toward greater emphasis on team coordination. Drone-based gameplay places relatively greater demands on operational control, spatial judgment, and context-sensitive decision making than on strength or endurance (Pfeiffer and Scaramuzza, 2021; Kaufmann et al., 2023). When this activity logic was paired with differentiated roles (e.g., pilot, strategy lead, observer), teams moved away from formats in which a single student dominated performance. Instead, a complementary, role-based team structure became more salient, such that collaboration focused on how each member contributed to shared play and decision making.

This account also connects with scholarship arguing that the educational significance of technology-based activities lies less in the equipment itself and more in how teachers integrate and contextualize digital tools within learning goals and instructional structures (Casey et al., 2017; Jastrow et al., 2022; Tohănean et al., 2025; Godhe et al., 2019). Accordingly, the strengthened collaboration and relational experiences observed in drone soccer can be interpreted less as an effect of technology *per se* and more as an outcome of instructional design that organized interdependence through role differentiation (Casey et al., 2017; Casey and Goodyear, 2015).

At the same time, the emergence of a “student-led” culture in School A should be interpreted with caution, as the researcher's dual role may have reinforced autonomy and collaboration. Future studies employing independent observers would further strengthen interpretive neutrality.

Team-based practice and strategy sharing during tournament preparation further illustrate how collaborative experiences formed in lessons extended into an external arena of practice. Students' comments that it “felt like we became a real team” suggest that drone soccer was experienced not merely as a short-term task but as a continuing relational experience. Overall, drone soccer highlights the potential of a PE model that leverages High-Tech elements while placing High-Touch relational experiences at the center of learning, integrating technology with human interaction. An important implication is that technology-integrated PE should be designed so that digital tools function as pedagogical mediators. Specifically, they should be used to structure interdependence and support communication, coordination, and role-based contribution rather than serving only as add-ons for demonstration or performance measurement (Casey et al., 2017; Jastrow et al., 2022; Tohănean et al., 2025).

### 4.3 Sustainability considerations and policy implications

The findings suggest that the drone soccer program was associated not only with changes in students' participation experiences but also with shifts in teachers' instructional perspectives during the 6-week implementation period. Although some teachers initially reported burden related to equipment operation and safety management, observing students' high engagement and visible participation changes appeared to prompt teachers to reconsider the educational value of technology-integrated physical education (PE). In particular, seeing students who had been passive in conventional PE participate actively through drone-based activities seemed to reframe technology as a pedagogical mediator that can reorganize participation structures and learning experiences (Casey et al., 2017; Jastrow et al., 2022; Tohănean et al., 2025).

From a sport psychology perspective, these shifts matter because teachers' technology-related self-efficacy, perceived competence, and pedagogical beliefs shape the quality of enactment and may influence whether new practices are maintained under routine constraints (Ertmer and Ottenbreit-Leftwich, 2010; Saiz-González et al., 2025). Professional learning for digital pedagogy has also been linked to teachers' confidence and wellbeing, which can affect what becomes feasible in everyday instruction (Shi et al., 2025). In particular, supporting teachers' capacity to design role-differentiated and participation-oriented lessons may be critical for sustaining inclusive practices within technology-integrated PE. In this study, teachers' intentions to establish drone soccer clubs after the formal program and to explore interdisciplinary or project-based lessons illustrate potential pathways through which a policy-supported program may catalyze continued instructional experimentation beyond the unit (Ertmer and Ottenbreit-Leftwich, 2010; Shi et al., 2025). However, long-term maintenance and institutionalization were not directly examined in this study and should be investigated through longitudinal research.

The findings also underscore that sustainability is likely to depend on implementation conditions that protect teachers' capacity and reduce practical and organizational load. District-level equipment provision and the deployment of specialist instructors may have alleviated burdens related to technical operation and safety supervision, thereby strengthening perceived feasibility and allowing teachers to focus on participation design and learning facilitation rather than logistics. Such conditions are consistent with evidence that resources, school-level support, and access to professional learning opportunities can mitigate common barriers to technology integration (Jastrow et al., 2022; Saiz-González et al., 2025). Sustaining technology-integrated PE may also require maintaining participation structures that broaden engagement for students who are underserved in conventional formats. Without structural and institutional support, innovative formats risk reverting to performance-centered logics that narrow inclusive participation conditions. Thus, policy efforts should not only provide technical resources but also protect pedagogical designs that sustain differentiated roles and multiple entry points to competence over time.

More broadly, sustainability requires more than one-time implementation. From a psychological perspective, sustainability in physical education is closely linked to meaningful participation, motivation, and learners' experiences, rather than mere continuation of activities (Baena-Morales et al., 2024). Prior work highlights multidimensional conditions such as stable funding, organizational capacity, partnerships, and systems for evaluation and feedback (Schell et al., 2013). In the context of school PE, sustainability may be conceptualized as the capacity to maintain and extend participation pathways beyond a single unit through clubs, interschool events, or other school-supported opportunities. This conceptualization extends sustainability beyond activity continuation to the preservation of diversified participation criteria within evolving school routines. The present cases suggest that district coordination combined with school-level commitment may create enabling conditions for extending technology-integrated activities beyond lessons; however, the durability of these pathways remains an open question that warrants longer-term follow-up.

An important implication is that meaningful implementation of technology-integrated PE should not rely solely on teachers' voluntary effort alone. Institutional and policy supports may be needed to stabilize practice, including routine equipment inspection and budget allocation, professional development that links technology with pedagogical design, flexibility for local adaptation within a shared curriculum, interschool events that provide authentic collective goals, and platforms for teacher case sharing and collaborative inquiry (Ertmer and Ottenbreit-Leftwich, 2010; Saiz-González et al., 2025; Schell et al., 2013). Overall, the study provides preliminary indications that policy-supported implementation may contribute to conditions associated with sustaining technology-integrated PE, while clearly recognizing that long-term sustainability outcomes were beyond the scope of the present 6-week study.

## 5 Conclusion

This comparative qualitative case study examined how a district-led drone soccer program, implemented as a technology-integrated physical education (PE) initiative, was associated with changes in middle school students' participation experiences, including those of sport-marginalized students, and how implementation conditions shaped school practice. The findings suggest that drone soccer (1) appeared to broaden participation by creating alternative pathways for perceived competence and contribution beyond conventional performance-centered PE, (2) may support relationship-centered learning by strengthening communication, coordination, and a sense of belonging through team-based High-Tech, High-Touch activity structures, and (3) was associated with greater potential for sustained enactment when teachers' instructional perspectives shifted alongside coordinated support from the education office and schools. Taken together, students' accounts suggest motivationally meaningful experiences characterized by heightened engagement, growing self-efficacy, and more positive peer relationships within the specific contexts examined.

These findings offer several practical implications. First, PE teachers may consider designing digital tools as pedagogical mediators that can reduce participation barriers and support key motivational processes through role diversification. In technology-based activities such as drone soccer, institutional recognition of diverse roles (e.g., piloting, strategy, recording, operations) may support sport-marginalized students' participation as core team members. Second, schools and district education offices may explore mid- to long-term support systems—including equipment maintenance, specialist staffing, teacher professional development, and interschool events—to facilitate implementation beyond individual teachers' effort. Third, curricular linkages across PE, technology education, and career education may help integrate such activities into coherent learning experiences that develop digital literacy, collaboration, and future-oriented motivation.

Overall, this case suggests that technology-integrated PE may create conditions conducive to inclusivity, relational learning, and continued enactment when pedagogical design is aligned with supportive implementation structures. Within the scope of this study, PE appears to function not only as physical activity provision but also as a context that may support students' motivational and socio-emotional experiences in digitally mediated schooling.

## 6 Limitations of the study

This qualitative case study examined a district-led drone soccer program in two middle schools (one girls' school and one boys' school) with a small number of student and teacher participants. While the design supported an in-depth, contextualized account of implementation and participation experiences, the findings should be interpreted in terms of transferability to similar contexts rather than statistical generalization. Future research could extend this work through additional cases across diverse regions and school types, or by using mixed-methods designs that integrate qualitative insights with complementary quantitative indicators.

The program was implemented over a relatively short period (approximately 6 weeks), and follow-up observation of post-program activities (e.g., tournament participation and subsequent club practice) was necessarily limited. As a result, this study could not fully examine longer-term influences on sustained engagement in PE, sport-related identity development, broader school adjustment, or career-oriented aspirations. Longitudinal research is needed to clarify how technology-integrated PE experiences are maintained, transferred, or attenuated over time.

Additionally, the study did not systematically assess physical activity intensity (e.g., moderate-to-vigorous physical activity), limiting conclusions regarding physiological outcomes. Future research may integrate objective activity measures alongside motivational and relational indicators to provide a more comprehensive evaluation of technology-integrated PE.

Because the researcher served as the supervising teacher at one participating school, the study benefited from close access and rich contextual knowledge, while also requiring careful attention to reflexivity. Credibility was strengthened through triangulation of interviews, observations, and documents, the inclusion of an interview with a teacher from another school, and peer debriefing and cross-checking of coding decisions. Finally, the analysis centered primarily on student and teacher perspectives; future studies should incorporate other stakeholders (e.g., administrators, parents, and education office officials) to examine multi-level conditions that support and sustain technology-integrated PE.

Although the discussion considered implications for inclusive PE, the study did not directly include students formally identified with disabilities. Therefore, conclusions regarding inclusive practice should be interpreted as conceptual extensions rather than empirical findings. Future research should examine how digitally mediated, role-differentiated PE activities operate within mixed-ability classrooms that include students with diverse functional profiles.

## Data Availability

The raw data supporting the conclusions of this article will be made available by the authors, without undue reservation.
